# Genotypic diversity effects on biomass production in native perennial bioenergy cropping systems

**DOI:** 10.1111/gcbb.12309

**Published:** 2016-01-10

**Authors:** Geoffrey P. Morris, Zhenbin Hu, Paul P. Grabowski, Justin O. Borevitz, Marie‐Anne de Graaff, R. Michael Miller, Julie D. Jastrow

**Affiliations:** ^1^ Department of AgronomyKansas State UniversityManhattanKS66506USA; ^2^ USDA‐ARS Dairy Forage Research CenterMadisonWI53706USA; ^3^ Research School of BiologyAustralian National UniversityActonACT2601Australia; ^4^ Department of Biological SciencesBoise State UniversityBoiseID83725USA; ^5^ Biosciences DivisionArgonne National LaboratoryArgonneIL60439USA

**Keywords:** big bluestem, biomass feedstock, cultivars, ecotype, fertilization, low‐input high‐diversity, polymorphism, switchgrass, tallgrass prairie, yield

## Abstract

The perennial grass species that are being developed as biomass feedstock crops harbor extensive genotypic diversity, but the effects of this diversity on biomass production are not well understood. We investigated the effects of genotypic diversity in switchgrass (*Panicum virgatum*) and big bluestem (*Andropogon gerardii*) on perennial biomass cropping systems in two experiments conducted over 2008–2014 at a 5.4‐ha fertile field site in northeastern Illinois, USA. We varied levels of switchgrass and big bluestem genotypic diversity using various local and nonlocal cultivars – under low or high species diversity, with or without nitrogen inputs – and quantified establishment, biomass yield, and biomass composition. In one experiment (‘agronomic trial’), we compared three switchgrass cultivars in monoculture to a switchgrass cultivar mixture and three different species mixtures, with or without N fertilization. In another experiment (‘diversity gradient’), we varied diversity levels in switchgrass and big bluestem (1, 2, 4, or 6 cultivars per plot), with one or two species per plot. In both experiments, cultivar mixtures produced yields equivalent to or greater than the best cultivars. In the agronomic trial, the three switchgrass mixture showed the highest production overall, though not significantly different than best cultivar monoculture. In the diversity gradient, genotypic mixtures had one‐third higher biomass production than the average monoculture, and none of the monocultures were significantly higher yielding than the average mixture. Year‐to‐year variation in yields was lowest in the three‐cultivar switchgrass mixtures and Cave‐In‐Rock (the southern Illinois cultivar) and also reduced in the mixture of switchgrass and big bluestem relative to the species monocultures. The effects of genotypic diversity on biomass composition were modest relative to the differences among species and genotypes. Our findings suggest that local genotypes can be included in biomass cropping systems without compromising yields and that genotypic mixtures could help provide high, stable yields of high‐quality biomass feedstocks.

## Introduction

In recent years, there has been great worldwide interest in the development of biomass cropping systems that could provide bioenergy feedstocks and reduce greenhouse gas emissions associated with fossil fuels. In the United States, Congress has mandated a major transition to lignocellulosic biofuels, which will require development of multiple new regionally appropriate biomass cropping systems (Downing *et al*., 2011). Among the top candidates for biomass energy crops are perennial grass species native to North American tallgrass prairies, including switchgrass (*Panicum virgatum*), big bluestem (*Andropogon gerardii*), and indiangrass (*Sorghastrum nutans*). These grasses share several characteristics that could be valuable for bioenergy production systems. Most notably they are capable of producing substantial yields of dry biomass with limited inputs and on land not suited to row cropping (Schmer *et al*., 2008; Griffith *et al*., 2011). In addition, bioenergy cropping systems based on native grasses may provide additional ecosystem services, such as carbon (C) sequestration (Tilman *et al*., 2006a; Gelfand *et al*., 2013) and wildlife habitat (Robertson *et al*., 2011).

Native perennial grasses have undergone some selection for forage, habitat restoration, and, increasingly, bioenergy uses, but most cultivars of these species remain essentially samples of the wild gene pool (Casler, 2012). As widespread wind‐pollinated species, prairie grasses harbor abundant genetic diversity. The ecotypic diversity and regional gene pools of switchgrass have been particularly well studied. In addition to classical work on continental clines (McMillan, 1959) and upland and lowland ecotypes (Porter, 1966), recent studies have identified phenotypic clinal variation across multiple axes (Casler, 2005; Casler *et al*., 2007) and many genetic subpopulations (Zalapa *et al*., 2010; Morris *et al*., 2011; Zhang *et al*., 2011; Grabowski *et al*., 2014). Though less well studied, big bluestem exhibits similar patterns of genotypic diversity at the phenotypic and genomic level (McMillan, 1956, 1961; Gray *et al*., 2014). As predominantly outcrossing species, switchgrass and big bluestem are both highly heterozygous. This abundant genotypic diversity provides useful genetic variance for crop improvement, but high heterozygosity and limited ability to inbreed can hinder many breeding approaches (Liu & Wu, 2012). Other candidate perennial biomass crops that originated in Asia, such as *Miscanthus* and *Saccharum* spp., exhibit similar patterns of diversity in their native ranges (Dillon *et al*., 2007; Kim *et al*., 2012).

In most crops, and most cropping systems, genotypic diversity has decreased over time, either directly due to selection for uniformity or indirectly due to genetic bottlenecks created by selection for other traits (Kingsbury, 2011). Direct selection on uniformity has been particularly central to the modern improvement of row crops, where uniform height and maturity is critical for efficient harvest and consistent quality. Accordingly, most commercial row crops are inbred varieties (e.g., wheat, soybean) or F1 hybrids of inbred lines (e.g., corn, sorghum) (Acquaah, 2012). By contrast, many perennial forage crops retain extensive genotypic diversity, either because uniformity is not necessary or desirable, or because the mating system precludes repeated inbreeding (e.g., obligate outcrossers). Given that the development of bioenergy cropping systems is at an early stage, it raises the question of what level of genotypic diversity should be targeted to increase yields in new perennial biomass crops. Moreover, as perennial biomass cropping systems are expected to provide a number of additional ecosystem services, genotypic diversity may influence trade‐offs or synergies among system outputs, such as feedstock yields, feedstock quality, soil C storage, or habitat for biodiversity conservation. While potential trade‐offs and synergies of species mixtures in perennial biomass cropping systems have been investigated (Tilman *et al*., 2006a; Adler *et al*., 2009; Griffith *et al*., 2011; Mangan *et al*., 2011; Jarchow & Liebman, 2012, 2013), the effects of genotype diversity mixtures (i.e., intraspecific diversity) are not well understood.

There are a number of reasons why genotypic diversity may be beneficial in perennial biomass cropping systems. Genetically, heterogeneous crop populations may buffer or hedge against temporal and spatial variability in the production environment (Allard & Bradshaw, 1964). This effect is thought to be important to smallholder production on marginal lands (Haussmann *et al*., 2012) and may be important for biomass cropping systems if production occurs on marginal lands, as expected (Downing *et al*., 2011; Uden *et al*., 2013). The most widespread use of designed genotypic mixtures is in multiline cultivars of cool‐season cereals, which can reduce the severity and spread of plant disease (Mundt, 2002). In native perennial systems, diverse mixtures often have higher productivity due to a sampling effect, the tendency of higher diversity systems to include at least one highly productive type (Fargione *et al*., 2007). Intraspecific diversity may also increase niche complementarity (Cook‐Patton *et al*., 2011), as is commonly seen with wild or cultivated interspecific mixtures (e.g., binary legume–grass mixtures) (Fargione *et al*., 2007; Nyfeler *et al*., 2009).

Many cultivars, species, and species mixtures have been investigated as potential lignocellulosic biomass crops because the choice of plant material has multiple impacts on the feasibility and sustainability of the biomass feedstock production system. For producers of biomass feedstock, high yield potential and efficient use of inputs will be critical for profitability (Boyer *et al*., 2012). Biomass cropping systems that have high net energy ratio (bioenergy output per unit input) yet produce low yields (biomass per unit area) may not be adopted because producer profitability is highly dependent on yields. Another concern for producers will be the opportunity cost of moving from a flexible annual cropping system to a perennial system that would require additional time to profitability (with little or no yields for several years following planting) (Uden *et al*., 2013). If native perennial bioenergy cropping systems are to be adopted, therefore, it is essential that yield potential be increased substantially, especially during the early years following planting.

For the biomass conversion industry, yields will also be important because ready access to low‐cost feedstock is required for plant profitability (Gan & Smith, 2011). Beyond feedstock access and cost, feedstock composition is important for many conversion technologies. For example, high‐digestibility low‐lignin feedstocks may be preferred for enzyme‐based conversion, while energy‐dense high‐lignin feedstock may be preferred for combustion‐based conversion. While ash is generally undesirable, particular elements (e.g., K, Na, Ca, S, and Cl) may be especially harmful for particular conversion technologies (Sims, 2003). The abundant natural variation of biomass composition among and within native perennial species (Sarath *et al*., 2008; Vogel *et al*., 2010; Zhang *et al*., 2015) provides an opportunity to optimize plant mixtures for a given fuelshed (e.g., 10s of km surrounding the bioenergy production facility) based on the conversion technology in use.

To investigate the effects of genotypic diversity on stand establishment, biomass yields, and biomass composition, we conducted two field experiments in northeastern Illinois over 2008–2014, where we varied switchgrass and big bluestem genotypic diversity, as well as other management variables. In the first experiment, we compared a switchgrass cultivar mixture to switchgrass cultivars grown in monoculture and multispecies mixtures, with and without nitrogen inputs. In the second experiment, we created a genotypic diversity gradient by varying cultivar numbers within switchgrass monocultures, big bluestem monocultures, and mixtures of switchgrass and big bluestem. Here, we describe the effects of varying genotypic diversity levels on stand establishment, biomass yield, and biomass composition and discuss the implications of these findings for the development of native perennial biomass cropping systems.

## Material and methods

### Site description and preparation

The experiments were conducted at the Fermilab National Environmental Research Park in Batavia, IL (N 41.8414, W 88.2297). Prior to the experiment (1971–2007), the 5.4 ha site was maintained as an old field dominated by cool‐season grasses – primarily smooth brome (*Bromus inermis*), quackgrass (*Agropyron repens*), and *Poa* species (O'Brien *et al*., 2013). The soil at the site is Grays silt loam (fine‐silty, mixed, superactive, mesic Mollic Oxyaquic Hapludalf), which is rated as prime farmland by US Department of Agriculture. Mean annual precipitation in the area is 920 mm with a mean temperature of 9.5°C. Monthly precipitation totals and monthly mean temperatures during the experiment were obtained from the National Climatic Data Center (http://www.ncdc.noaa.gov/) for Chicago West DuPage Airport weather station (USW00094892), located approximately 8 km north of our field site. In fall 2007, standing vegetation at the site was removed by application of the broad‐spectrum herbicide glyphosate followed by burning of the dead vegetation. Subsequent regrowth in spring 2008 was treated again with glyphosate twice before planting. Switchgrass monocultures were seeded at 6.7 kg pure live seed (PLS) ha^−1^ (~575 seeds m^−2^), which is within the seeding rate range (5.6–7.3 kg PLS ha^−1^) recommended by the US Department of Agriculture (USDA) Natural Resources Conservation Service (NRCS) for switchgrass feedstock production in the Midwest. Seeding rates in the plots that included other species were adjusted to achieve the same total seeding rate as the switchgrass monocultures (~575 seeds m^−2^), with the proportion of each species and ecotype given in Table 1. The monocultures and mixtures are described in detail below. All sown species are perennials and native to the region (Swink & Wilhelm, 1994).

**Table 1:** Plant materials and sown plant treatments for the agronomic trial. Values in parenthesis indicate the sown cultivar composition (based on % of sown seeds) for switchgrass and big bluestem, or sown species composition for the forbs. The plant treatments are Kanlow switchgrass (KA), Cave‐In‐Rock switchgrass (CR), Southlow switchgrass (SL), a three switchgrass cultivar mixture (SG), a big bluestem plus switchgrass mixture (BB), a Canada wildrye plus switchgrass mixture (CW), and a prairie mixture (PR)

Species	Common name	Type	Cultivar	Plant mixture treatments (sown %)						
				KA	CR	SL	SG	BB	CW	PR
*Panicum virgatum*	Switchgrass	C4 grass		100%	100%	100%	100%	50%	60%	20%
			Kanlow	(100%)			(33%)	(17%)	(20%)	(7%)
			Cave‐In‐Rock		(100%)		(33%)	(17%)	(20%)	(7%)
			Southlow			(100%)	(33%)	(17%)	(20%)	(7%)
*Andropogon gerardii*	Big bluestem	C4 grass						50%		20%
			Rountree					(17%)		(7%)
			Epic					(17%)		(7%)
			Southlow					(17%)		(7%)
*Sorghastrum nutans*	Indiangrass	C4 grass								20%
*Elymus canadensis*	Canada wildrye	C3 grass							40%	20%
		Forbs								20%
*Desmodium canadense*	Showy tick trefoil	Legume								(2.50%)
*Lespedeza capitata*	Round‐headed bush clover	Legume								(2.50%)
*Dalea purpurea*	Purple prairie clover	Legume								(2.50%)
*Aster nova‐angliae*	New England aster	Composite								(2.50%)
*Coreopsis tripteris*	Tall tickseed	Composite								(2.50%)
*Heliopsis helianthoides*	Smooth oxeye	Composite								(2.50%)
*Ratibida pinnata*	Yellow coneflower	Composite								(2.50%)
*Veronicastrum virginicum*	Culver's root	Other forb								(2.50%)

### Switchgrass and big bluestem germplasm

The switchgrass and big bluestem cultivars were chosen to represent a wide range of diversity in each species. The switchgrass germplasm used consisted of the following, listed in order of most northern to most southern origin. Dacotah is derived from progeny of a single plant from North Dakota and originates from the most northern and driest location (380 mm) among the selected cultivars (Barker *et al*., 1990). Forestburg is a composite of four accessions from eastern South Dakota (Barker *et al*., 1988). Sunburst is derived from multiple plants from one county in southeastern South Dakota, which were subjected to three cycles of selection for vigor, leafiness, and seed weight (Boe & Ross, 1998). Southlow switchgrass is an ecopool from southern Lower Michigan, a composite of germplasm from 11 native stands crossed and increased with no purposeful selection (Durling *et al*., 2008). Cave‐In‐Rock was developed from germplasm originating at a native stand in southern Illinois (Hanson, 1972). Blackwell originates from a single plant collected at an upland site in northern Oklahoma and was tested in northeastern Kansas prior to release (Hanson, 1972). Kanlow originates from germplasm collected at a lowland site in central Oklahoma and was subject to selection for leafiness, vigor, and late season greenness in northeastern Kansas prior to release (Hanson, 1972).

The big bluestem germplasm used was the following. Southlow big bluestem is an ecopool from southern Lower Michigan, a composite of germplasm from 22 native stands crossed and increased with no purposeful selection (Durling *et al*., 2007). Champ is a hybrid of sand bluestem (*Andropogon hallii*) from Nebraska and big bluestem from multiple sites in Nebraska and Iowa (Newell, 1968a). Pawnee was developed from germplasm originating from a county in southeastern Nebraska and was selected for several generations in Nebraska (Newell, 1968b). Bonanza is a derivative of Pawnee, selected for three generations for forage yield and digestibility across three sites in Nebraska (Vogel *et al*., 2006). Rountree originates from a native stand in western Iowa and was selected and increased in eastern Missouri (Alderson & Sharp, 1994). Epic originates from a site in western Arkansas and was selected and increased in eastern Missouri (USDA‐NRCS, 2013). Suther originates from a native stand in central North Carolina and was tested and increased in New Jersey (Davis *et al*., 2002).

### Experimental design for agronomic trial

The agronomic trial has seven plant treatments in three randomized complete blocks with two split‐plot fertilization treatments, for a total of 42 plots. Each plot is 36 m × 20 m. The plant treatments (Table 1) consist of three switchgrass monocultures [the lowland cultivar Kanlow (KA), upland cultivar Cave‐In‐Rock (CR), and regional ecopool Southlow (SL)]; a switchgrass mixture with all three varieties (SG); binary mixtures of switchgrass with big bluestem (BB), or switchgrass with Canada wildrye (CW); and a 12‐species prairie mixture that includes the preceding grasses along with other grasses and forbs native to the region (PR). Plots are separated by 2 m (east–west) or 4 m (north–south) alleys sown with a low‐stature fescue mix (*Festuca* spp.) and replicate plot treatments are assigned to northern, central, and southern blocks. This experiment was managed using conventional agricultural techniques. Plant treatments were sown in June 2008, by no‐till drill‐seeding using a native seed drill at a depth of ~0.5 cm in ~20 cm rows. In the fertilized plots, granular urea (46‐0‐0) was applied annually during the first week of June (beginning in 2009) with a hand broadcast spreader at a rate of 67 kg N ha^−1^, within the recommended range for switchgrass feedstock production (50–100 kg N ha^−1^) (USDA‐Natural Resources Conservation Service, 2009). The weeds were controlled by broadcast application of Milestone (aminopyralid) and Garlon (triclopyr) broad‐leaf herbicides in 2009 (except the PR mixture, which includes broad‐leaf species). In 2010, weed control included spot application of Milestone and Garlon to several patches of crown vetch (*Securigera varia*), dogwood (*Cornus* sp.), and oxeye daisy (*Leucanthemum vulgare*) and spot application of Round‐Up (glyphosate) to reed canary grass (*Phalaris arundinacea*).

### Experimental design for diversity gradient

The diversity gradient consists of four levels of cultivar diversity (1, 2, 4, 6) and three species treatments (switchgrass, big bluestem, or switchgrass and big bluestem mixture) for a total of 164 plots (3 m × 2 m each). Each of seven cultivars of switchgrass and seven cultivars of big bluestem was grown in four replicate monoculture plots (2 species × 7 cultivars × 4 replicates = 56 plots). For the single‐species cultivar mixtures (‘Swi’, ‘Big’), there are 12 replicates at each cultivar diversity level (2, 4, 6), with cultivars sampled randomly and seeded at equal rates (2 species × 3 diversity levels × 12 replicates = 72 plots). For the mixed‐species cultivar mixtures (‘Mix’), there are 12 replicates at each cultivar diversity level with cultivars sampled randomly at a 1 : 1 ratio of switchgrass and big bluestem (3 diversity levels × 12 replicates = 36 plots). Alternating rows are separated by 1 m east–west alleys, and the experiment is surrounded and bisected by larger alleys from the Agronomic trial. Replicates are blocked according to exposure to alleys. Seeds were hand‐sown into ~0.5‐cm‐deep furrows in 20 cm rows. Two months postsowing, all plots showed germination of the sown species (i.e., switchgrass, big bluestem, or both). The plots were hand‐weeded in the spring from 2008 to 2010, and treated with broad‐leaf herbicide (Milestone and Garlon) in 2009.

### Biomass yield measurements

**Figure 1:**
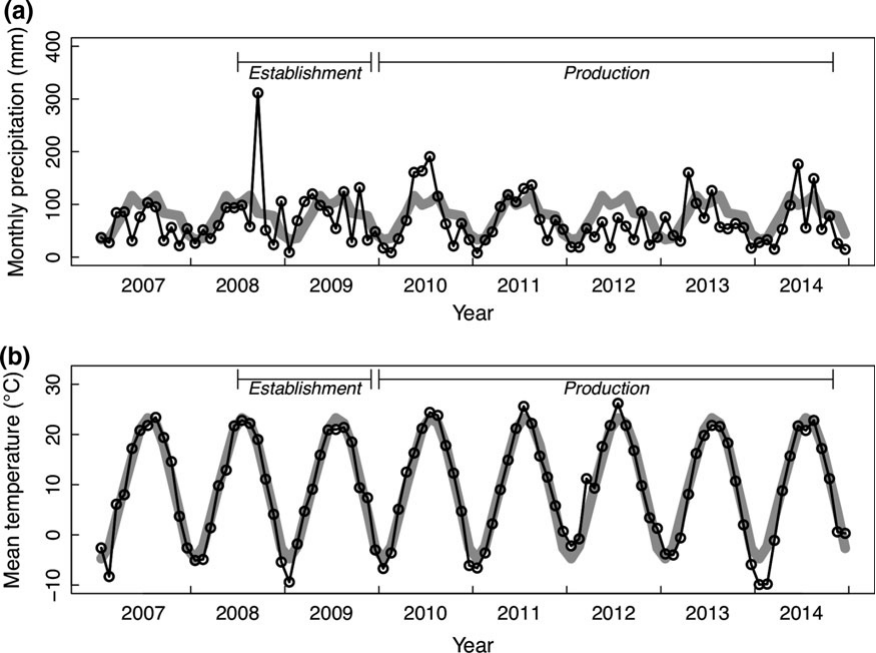
Monthly precipitation (a) and mean monthly temperature conditions (b) during the experiment. The 20‐year averages (1981–2001) are shown with the gray lines. Data are from 8 km north of the field site.

To account for the time it takes for native perennial systems to establish, we define the first 2 years of the experiment as establishment years (2008–2010) and the subsequent 5 years (2009–2014) as production years (Fig. 1). In the first establishment year (November 2008) and first production year (September 2010), the standing crop of aboveground biomass in the agronomic trial was estimated for sown species and weeds using 0.5‐m^2^ circular quadrats (Kennedy, 1972). Four quadrats were randomly placed in each plot, and all stems within the area were clipped to 2–4 cm aboveground level. In years 2009–2014, aboveground biomass was harvested at ~15 cm aboveground level (USDA‐Natural Resources Conservation Service, 2009) after a killing frost (early‐ to mid‐November) using standard commercial hay machinery. Each plot was harvested individually and produced one or more round bales (of variable size) per plot, which were weighed with a hanging scale. For each diversity gradient plot, in years 2009–2014, the entire plot was harvested with a string trimmer just aboveground level, and the biomass was collected, so that essentially all aboveground biomass was removed. Moisture content of biomass from each bale/plot was estimated from a subsample taken immediately prior to baling or weighing, and all reported yields have been adjusted to a dry‐weight basis (65°C). The effects of plant treatment and nitrogen fertilization on biomass yields, stand establishment, and plant composition in the agronomic trial were determined by repeated measures split‐plot anova implemented with aov in the R statistical computing environment (R Core Team, 2014). The *P*‐values given in the text for significant factors are from minimal adequate models, obtained after sequentially dropping nonsignificant (*P *>* *0.05) factors from the model (Crawley, 2012).

### Genetic diversity estimates

Published Illumina short‐read sequence data for 123 switchgrass genotypes along with barcode multiplexing information (Grabowski *et al*., 2014) were downloaded from the NCBI Sequence Read Archive (Accession: PRJNA252891) and Dryad Digital Repository (doi:10.5061/dryad.k77nh), respectively. The raw data were demultiplexed with ea‐utils.1.1.2‐537 (Aronesty, 2013), and adapters were removed with the FASTX‐Toolkit (http://hannonlab.cshl.edu/fastx_toolkit/). The individual fastq files were aligned on the reference genome with BWA (Li & Durbin, 2009), and single nucleotide polymorphisms (SNPs) were called with SAMtools (Li *et al*., 2009) with default settings. The genetic distance of individuals and the neighbor‐joining (NJ) tree was calculated with TASSEL 4 (Bradbury *et al*., 2007), and the NJ tree was visualized with MEGA 6 (Tamura *et al*., 2013). Nucleotide diversity was calculated with 2*p*(1−*p*), where *p* represents the allele frequency. The allele frequencies were calculated with VCFtools (Danecek *et al*., 2011), and the figure was drawn with R (R Core Team, 2014).

### Biomass composition analysis

Subsamples of the whole‐plot harvested biomass were taken for all plots (both agronomic trial and diversity gradient) in 2012. The samples were dried at 65°C and sent to a commercial plant testing laboratory, Dairyland Laboratories Inc. (Arcadia, WI, USA), for grinding in a cyclone mill and compositional analysis using wet chemistry and near‐infrared reflectance spectroscopy (NIRS). Mineral analysis (Ca, P, K, Mg, and S) was carried out using wet chemistry methods (AOAC Official Method 953.01). NIRS estimates of lignin, hemicellulose, cellulose, fat, sugar, ash, and nitrogen were performed on a FOSS 5000 using calibrations developed in WinISI (FOSS North America, Eden Prairie, MN). Calibrations were based on wet chemistry (AOAC Official Methods 973.18, 2002.04, 920.39, 942.05, 990.03) from a worldwide panel of mixed hay samples (*N* ranging from 355 to 7140), with an *r* ^2^ based on validation samples ranging from 0.73 to 0.99. All values are provided on a dry matter basis.

## Results

### Precipitation and temperature conditions

Stand establishment and biomass production are expected to be influenced by precipitation and temperature. The monthly precipitation and average monthly temperatures during the experiment are presented along with 20‐year averages for comparison (Fig. 1). The first establishment year (2008) and first production year (2010) were unusually wet, with 2008 being the wettest year on record (1871–2014) for the Chicago area (US Department of Commerce, 2015). The third production year coincided with the historic drought of 2012 and total precipitation in the 6 months preceding harvest was just 56% of the 20‐year average. The 2012 drought year also had unusually high temperatures in July, 3°C above the 20‐year average for the month. The first winter postplanting was unusually cold, with January 2009 temperatures 5°C below the 20‐year average. The winter prior to the 2014 production year was also unusually cold with January and February 2014 temperatures 5°C and 7°C below the 20‐year averages, respectively.

### Stand establishment

**Figure 2:**
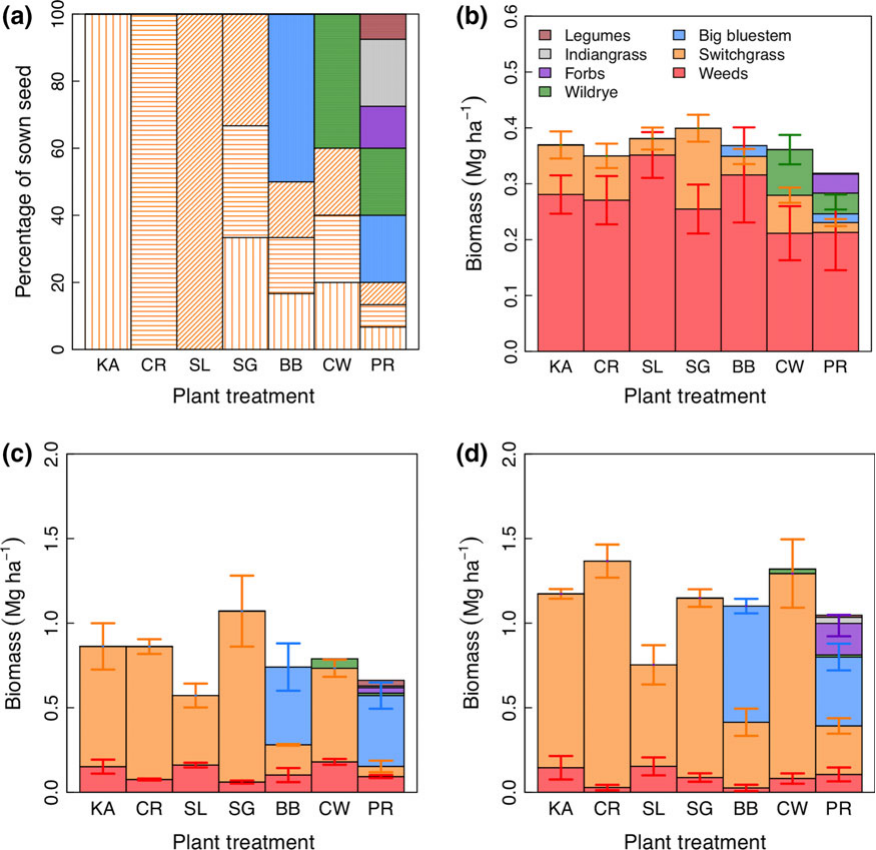
Plant establishment and plot composition based on peak aboveground biomass in the agronomic trial. Shown are the percentage of sown seed in each plant treatment (a), the observed biomass composition (mean ± standard error) in the establishment year 2008 (b), and the first production year 2010, unfertilized (c), and fertilized (d). The plant treatments are Kanlow switchgrass (KA), Cave‐In‐Rock switchgrass (CR), Southlow switchgrass (SL), a three switchgrass cultivar mixture (SG), a big bluestem plus switchgrass mixture (BB), a Canada wildrye plus switchgrass mixture (CW), and a prairie mixture (PR). Shading in (a) designates the three switchgrass cultivars, Kanlow (vertical), Cave‐In‐Rock (horizontal), and Southlow (diagonal). For 2008, (b) the data are averaged over all plots in a given plant treatment because no fertilizer had been applied at that point. ‘Forbs’ refers to all nonlegume sown forbs.

To characterize any differences in stand establishment among the treatments in the agronomic trial, we compared the sown plant composition (Fig. 2a) to the observed plant composition in the first establishment year (2008) (Fig. 2b) and first production year (2010) (Fig. 2c,d). In 2008, the average aboveground standing crop was 0.4 Mg ha^−1^, of which only 25% was accounted for by sown plant species. No significant differences were observed among the plant treatments either for the total amount of biomass (*P *=* *0.11) or for the proportion of weed biomass (*P *=* *0.24). By 2010, the average biomass was 9.6 Mg ha^−1^ and the proportion of biomass accounted for by sown plant species increased to 90%, on average. Here, there were significant differences in the proportion of sown vs. weed biomass among plant treatments (*P *<* *0.01), with Southlow switchgrass showing poor establishment compared to most other plant treatments (~65% of biomass; Tukey's HSD *P *<* *0.03). In addition, the unfertilized plots showed a significantly higher proportion of weed biomass compared to the fertilized plots (20% vs. 10%; *P *<* *0.01). The total biomass (sown species plus weeds) was 30% less in the unfertilized plots compared to the fertilized plots (*P* < 0.0001). Overall, total biomass was approximately equal in all plant treatments except Southlow switchgrass, which had 34% less biomass than the others (*P *<* *0.01). No significant plant treatment by fertilizer interactions on plant composition or total biomass was observed during stand establishment or the first production year.

### Biomass yields in genotypic and species mixtures in the agronomic trial

**Figure 3:**
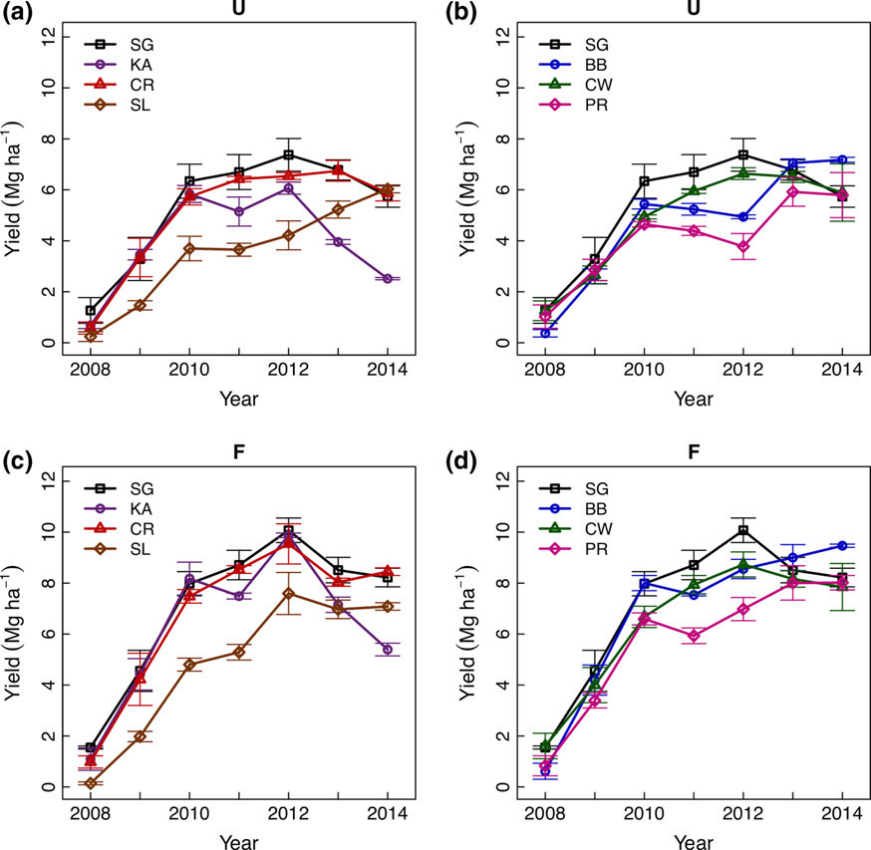
Biomass yields over 7 years from 14 perennial cropping systems. Plotted are the means ± standard error (*n* = 3) of dry‐weight yields for whole‐plot harvestable biomass (or for 2008, an estimate based on quadrat data). The plant treatments are Kanlow switchgrass (KA), Cave‐In‐Rock switchgrass (CR), Southlow switchgrass (SL), a three switchgrass cultivar mixture (SG), a big bluestem plus switchgrass mixture (BB), a Canada wildrye plus switchgrass mixture (CW), and a prairie mixture (PR). The top panels (a and b) are results from unfertilized (‘U’) plots, while the bottom panels (c and d) are from fertilized (‘F’) plots. Averaged over the five production years (2010–2014) and across two fertilization levels, the mixture of three switchgrass cultivars (SG) had the highest yield (though not significantly higher than BB, CR, and CW).

Biomass yields were determined in 2009–2014 by harvesting entire plots after plant senescence (Fig. 3). By the third year of the experiment (2010), the total yield across all plots was statistically indistinguishable from the total yield in later years, supporting the definition of 2008–2009 as establishment years and 2010–2014 as production years. Averaging over the five production years, plant treatment and fertilization effects were both highly significant (*P *<* *0.0001). Block effects were not observed (*P *=* *0.44) and are not considered further here. The mean yield increase due to nitrogen fertilization (67 kg N ha^−1^) was 2.2 Mg ha^−1^, and no interaction was observed between fertilization and plant treatment (*P *=* *0.55). The switchgrass cultivar mixture (SG) produced the greatest yield overall, averaging 8.7 Mg ha^−1^ across 5 years when fertilized and 6.6 Mg ha^−1^ when unfertilized. Average yields were similar for the Cave‐In‐Rock cultivar monoculture (CR) and the binary species mixtures of switchgrass with big bluestem (BB) and switchgrass with Canada wildrye (CW) (Tukey's HSD test *P *>* *0.05). In contrast, we observed lower average yields for the Kanlow and Southlow cultivar monocultures (KA and SL) and the prairie mixture (PR). Overall, the yields from the switchgrass‐only treatments (SG, KA, CR, and SL) were no greater or less than the mixtures with other species (CW, BB, and PR) (*P *=* *0.79).

Across the production years, the yield trends were significantly different among plant treatments (ancova *P *<* *0.0001). Comparing each plant treatment to Cave‐In‐Rock, the switchgrass cultivar most likely *a priori* to be well adapted and high yielding in this region, some plant treatments showed significant yield increases over the production years, while others showed yield decreases. An increasing yield over the production years was observed in the Southlow cultivar (SL, 0.6 Mg ha^−1^ yr^−1^, *P *=* *0.02) and the binary mixture of switchgrass and big bluestem (BB, 0.5 Mg ha^−1^ yr^−1^, *P *=* *0.02). In contrast, a trend of decreasing yield was observed in the Kanlow cultivar (KA, −0.7 Mg ha^−1^ yr^−1^, *P *<* *0.0001). There was no evidence of fertilization by year interaction or a three‐way interaction of fertilization with plant treatment over years (*P *=* *0.98).

### Biomass yields over the genotypic diversity gradient

**Figure 4:**
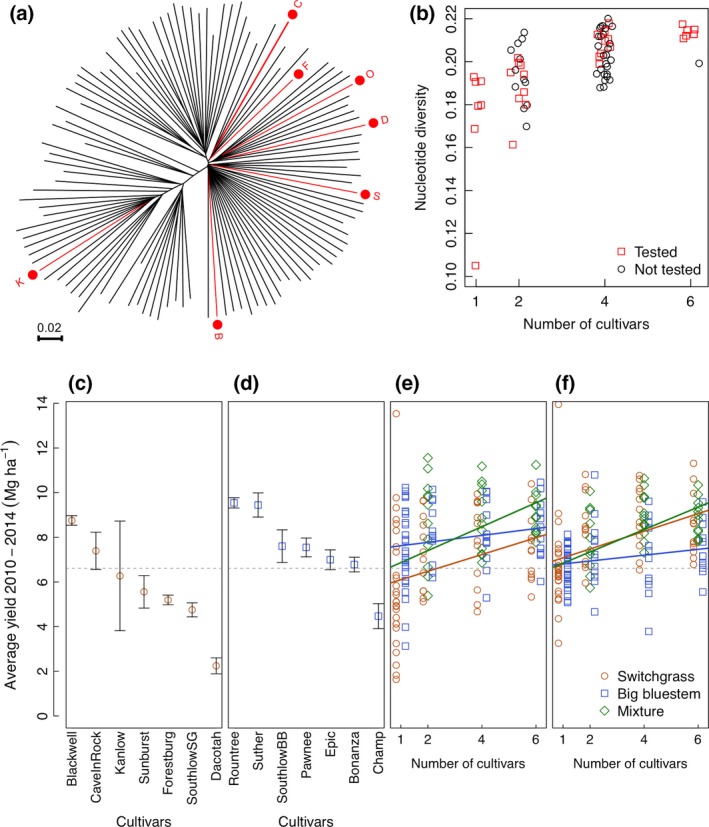
Biomass yields along a genotypic diversity gradient for switchgrass and big bluestem. (a) Genetic variation of the seven switchgrass cultivars used in the experiment relative to the continent‐wide diversity of switchgrass, estimated from single nucleotide polymorphism, and plotted on a neighbor‐joining tree (K = Kanlow, B = Blackwell, S = Sunburst, D = Dacotah, O = Southlow, F = Forestburg, C = Cave‐In‐Rock). (b) The nucleotide diversity (average pairwise difference) in switchgrass monocultures and cultivar mixtures used in the field experiment (red, ‘Tested’) and other possible mixtures of the seven cultivars (black, ‘Not Tested’). (c–f) The mean yields over five production years (± standard error) of the switchgrass (c) and big bluestem (d) cultivar monocultures are compared to those of mixtures (e and f), with the observed mixture yields (e) or the ‘scaled’ mixture yields (f), which are corrected for the expected yield of each mixture based on the average monoculture yields of the component cultivars. The cultivar monocultures (c and d) are sorted in order of decreasing mean yield. The dotted line indicates the global mean across plant treatments.

To better understand genotypic diversity effects on biomass production, we carried out a parallel experiment on a diversity gradient with four levels of cultivar richness (1, 2, 4, or 6) and three species treatments (switchgrass, big bluestem, or a binary mixture of the two). The seven switchgrass and seven big bluestem cultivars used in this experiment were chosen to represent a broad sample of the geographic range and genetic variation for each species (see Materials and Methods). For the switchgrass treatments, we estimated the sown genotypic diversity based on nucleotide diversity from genotyping‐by‐sequencing data and found significantly higher polymorphism in the cultivar mixtures (*P *<* *0.0001, *r* ^2^ = 0.33; Fig. 4a,b). There were large differences in biomass yield among the 14 cultivars when grown in monoculture (Fig. 4c,d). (Note that biomass yields from the diversity gradient plots are presented as Mg ha^−1^ yr^−1^ for comparison with agronomic trial results, not to provide absolute yield estimates). When grown in cultivar monocultures, the big bluestem cultivars yielded somewhat more than the switchgrass cultivars, averaging +1.7 Mg ha^−1^ yr^−1^ over the production years of 2010–2014 (*P *<* *0.01). Among the cultivar monocultures, the species effect (switchgrass vs. big bluestem) explains 12% of the variation, while cultivar (nested within species) explains 43% of the variation (*P*_sp _< 0.001; *P*_sp:cv_ < 0.0001).

To test the effects of genotypic diversity on biomass production, we compared the cultivar monocultures described above (*n* = 56) to randomly chosen mixtures of the same cultivars (2, 4, or 6 cultivars per plot; *n* = 108). Among the cultivar mixture plots, two‐thirds were sown with only switchgrass or only big bluestem, while one‐third were sown with 1 : 1 mixtures of the two species (e.g., three switchgrass cultivars and three big bluestem cultivars in a six cultivar plot). Averaging yields over the production years (2010–2014), we observed a significant positive relationship between yield and number of cultivars (Fig. 4e,f; *P *=* *0.02). Comparing yields of the cultivar monocultures to the genotypic/species mixtures, we see a higher average yield (31% for switchgrass, 9.5% for big bluestem, 34% for the species mixture) in the mixtures (2, 4, or 6 cultivars) than the average yield of the cultivars grown in monoculture (*P *<* *0.01). Only one switchgrass cultivar monoculture, Blackwell, had a higher mean yield than the switchgrass cultivar mixtures (8.1 vs. 7.5 Mg ha^−1^) although this was not significant (*P *=* *0.15). Two big bluestem cultivar monocultures, Rountree and Suther, had higher mean yield than the big bluestem cultivar mixtures (9.5 and 9.4 vs. 8.2 Mg ha^−1^), but the differences were again not significant (*P *=* *0.1).

### Year‐to‐year stability of biomass yields

**Figure 5:**
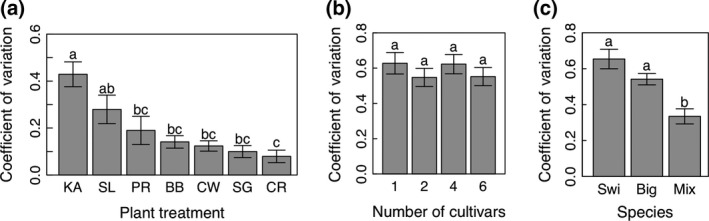
Differences in year‐to‐year variation for yield among plant treatments across the production years (2010–2014). Significant differences among plant treatments are observed for the agronomic trial (a). The plant treatments are Kanlow switchgrass (KA), Cave‐In‐Rock switchgrass (CR), Southlow switchgrass (SL), a three switchgrass cultivar mixture (SG), a big bluestem plus switchgrass mixture (BB), a Canada wildrye plus switchgrass mixture (CW), and a prairie mixture (PR). For the diversity gradient, there is no significant difference by number of cultivars (b), but there is a difference by species treatment (c) (‘Swi’ = switchgrass, ‘Big’ = big bluestem, and ‘Mix’ = mixtures of the two species).

In addition to mean biomass yields, we also considered the differences in year‐to‐year stability of yields, estimated as the coefficient of variation for yield across years. In the agronomic trial, the plant treatments differ significantly in the year‐to‐year variation in yield over the production years (Fig. 5a; *P *<* *0.0001). The fertilized and unfertilized treatments did not have significantly different coefficient of variation for yield (*P *=* *0.29). Kanlow and Southlow had the highest coefficients of variation while Cave‐in‐Rock and the three‐cultivar switchgrass mixture had lower variation (Fig. 5a). Overall, the year‐to‐year coefficient of variation was slightly higher in the switchgrass cultivar monocultures than the switchgrass cultivar mixture (0.26 vs. 0.1; *P *<* *0.05). In the diversity gradient experiment, no significant reduction in year‐to‐year variation was observed in the plots with higher genotypic diversity Fig. 5b; *P *=* *0.11). However, reduced year‐to‐year variation was observed in the plots with both switchgrass and big bluestem as compared to the plots with only one of the species (Fig. 5c; *P *<* *0.0001).

### Composition of harvested biomass

The final aspect of the cropping systems we evaluated was the composition of the harvested biomass. To identify differences among treatments, we estimated the content of the structural components (lignin, cellulose, hemicellulose), nonstructural organic compounds (sugar, nonfiber carbohydrates, fats), and minerals (N, P, K, S, Mg, Na, Cl) from all plots in both experiments in 2012, midway through the production years. Here, we highlight a few of these compositional effects from each experiment. In the agronomic trial, we observed significant plant treatment differences and fertilization effects for several constituents, and plant treatment by fertilization interaction in a few cases (Table 2). The differences in lignin content among plant treatments were highly significant (*P *<* *0.0001), with higher lignin content in the prairie mixture (PR; Tukey's *P *<* *0.01) and lower lignin content in Kanlow switchgrass (KA; Tukey's *P *<* *0.01). Cellulose content was significantly lower in the big bluestem–switchgrass mixture (BB) and higher in switchgrass biomass (Tukey's *P *<* *0.0001). N fertilization leads to a significant, but small, increase in biomass N content from 0.59% to 0.66% (*P *<* *0.001). There was no evidence of differing N content among plant treatments, but there was significant interaction with fertilization (*P *=* *0.047) due to higher N levels in the fertilized big bluestem‐switchgrass mixture (BB). The biomass P content is significantly lower with N fertilization (*P *<* *0.0001), but there were no differences in K content among treatments. The only significant difference in ash content was the higher ash content of Southlow vs. Kanlow switchgrass (7.0% vs. 5.4%; *P *=* *0.02).

**Table 2:** Biomass composition by plant treatment (top; *n* = 6) or N fertilization treatment (bottom; *n* = 21). Values that are significantly different (Tukey's HSD, *α * =  0.05) among cultivars or diversity levels are indicated by a different letter

	Lignin	Cell	Hemi	Sugar	NFC	Fat	Ash	N	P	K	Mg	S	Ca	Na	Cl
Plant treatment															
KA	7.5c	47.7a	30.2a	5.0a	3.5c	1.6a	5.4b	0.62a	0.037a	0.16a	0.17a	0.058a	0.21d	103.0a	0.058bc
CR	8.1b	46.5a	28.6ab	4.6ab	5.7abc	1.4ab	5.9ab	0.58a	0.048a	0.19a	0.16a	0.047a	0.33c	78.8a	0.083a
SL	8.3ab	46.8a	28.4b	3.4c	4.0bc	1.3b	7.0a	0.63a	0.060a	0.17a	0.15a	0.050a	0.42ab	83.8a	0.073ab
SG	8.1ab	46.5a	28.4b	4.4ab	5.1abc	1.4ab	6.4ab	0.63a	0.045a	0.20a	0.16a	0.057a	0.36bc	82.0a	0.070abc
BB	7.9bc	44.4b	29.2ab	4.1b	6.7a	1.5ab	6.0ab	0.67a	0.048a	0.15a	0.15a	0.043a	0.36bc	77.8a	0.052c
CW	8.0bc	45.9ab	28.7ab	4.6ab	5.8abc	1.5ab	6.1ab	0.60a	0.047a	0.19a	0.16a	0.047a	0.34c	88.2a	0.068abc
PR	8.7a	46.4ab	26.4c	3.9bc	6.2ab	1.5ab	6.6ab	0.65a	0.045a	0.18a	0.15a	0.045a	0.44a	81.2a	0.058bc
N Fert.															
Unfert.	7.9b	45.8b	28.7a	4.5a	5.8a	1.5a	6.4a	0.59b	0.057a	0.18a	0.15b	0.050a	0.35a	81.7a	0.069a
Fertilized	8.3a	46.8a	28.4a	4.1a	4.8a	1.4b	6.0a	0.66a	0.037b	0.17a	0.17a	0.050a	0.36a	88.2a	0.064a

Cell, cellulose; Hemi, hemicellulose; NFC, nonfiber carbohydrate.

In the genotypic diversity gradient, switchgrass biomass had lower estimated cellulose content (44.6%) than big bluestem (46.8%; Tukey's *P *<* *0.0001) and the mixtures of big bluestem and switchgrass (45.5%; Tukey's *P *<* *0.001). Conversely, switchgrass had higher estimated sugar content (4.9%) than big bluestem (4.1%; Tukey's *P *<* *0.0001) and the two species mixture (4.5%; Tukey's *P *=* *0.001). The level of cultivar diversity had no significant effects on the organic compound content, and little effect on mineral composition.

## Discussion

In this study, we characterized the effects of plant genotypic and species diversity on establishment, yields, and biomass composition and consider some implications for production, conversion and environmental impacts. Overall, we found genotype mixtures performed well in terms of yield (Figs 3, 4 and 5). Given that this was true even when some of the constituent cultivars performed poorly in monoculture, this suggests that there is little risk of low yields in using genotypic mixtures as long as some adapted material is included. We see that use of genotypic diversity can stabilize yields (as seen with SG), but so can the choice of a high‐yielding and well‐adapted cultivar monoculture (in this case, Cave‐In‐Rock) (Fig. 5a). These observations are consistent with a sampling effect because the yields of the genotypic mixtures are not transgressive (Fargione *et al*., 2007). In more marginal production environments, where temporal and spatial heterogeneity is proportionally more important, there may be greater benefits of genotypic diversity (Haussmann *et al*., 2012). Further studies of genotypic mixtures of biomass species grown in marginal production environments (e.g., drought‐prone or nutrient deficient soils) will be needed to evaluate this hypothesis. In addition, future studies of targeted genotypic mixtures (i.e., selected for complementary traits or high yield potential) might reveal benefits of genotypic diversity not observed in the random genotype mixtures we studied here.

We did not estimate the abundances of the three switchgrass cultivars separately in the cultivar mixture, but given biomass composition estimates, it appears that all three cultivars were well represented. For example, the higher ash content of SG and SL treatments than KA and CR (Table 2) suggests that Southlow was abundant in the three‐cultivar mixture by 2012 despite its slow establishment in monoculture. These findings show that a local ecopool cultivar with high genotypic diversity can meet or exceed the biomass yields from cultivars with high expected yields when production is considered over a longer time scale.

While high average yields are important, the stability of yields may be equally important in marginal production systems with localized value chains. For instance, in drier years, crops with high yield potential but drought sensitivity could fail to meet the year‐round feedstock supplies required by the bioconversion plants (Uden *et al*., 2013). In row cropping and native grassland systems, mixtures of genotypes and species have been shown to stabilize productivity (Smithson & Lenné, 1996; Tilman *et al*., 2006b) and thus may be expected to limit yield losses to environmental stresses, such as cold spells or drought. In fact, in this experiment, the highest overall biomass yields were observed in the three switchgrass mixture (SG) during the drought year of 2012 (true for both fertilized and unfertilized treatments; Fig. 3). In contrast, the 2012 corn yields in the county were about 20% below the 2011 yields and the long‐term yield trend (USDA‐NASS, 2013). The ability of diverse native perennial biomass cropping systems to produce high yields when row crop (e.g., corn or sorghum) stover yields are low may be especially valuable for ensuring stable feedstock supplies in a fuel shed despite annual variability (Uden *et al*., 2013).

Diverse species mixtures are commonly used in forage systems – especially mixtures of legumes and cool‐season grasses – and these polycultures often substantially outperform forage monocultures (Picasso *et al*., 2008). As most of the weed species at our field site were cool‐season species, we hypothesized that the plant treatments that included cool‐season species (the wildrye with switchgrass and the 12‐species prairie mixture) could reduce weed biomass during establishment. However, there was no evidence that the cool‐season species we included were able to out compete the cool‐season weeds. Instead, vigorous growth in the establishment year by improved cultivars seemed to be most associated with weed suppression. Further studies with other cool‐season native species will be needed to determine whether other warm + cool‐season native grass mixtures are able to provide improved yields.

While interactions between species diversity and fertilization have been investigated, interactions between genotypic diversity and fertilization have been less studied (Jarchow & Liebman, 2013). In this study, there was no evidence that switchgrass monocultures responded more to N fertilization than the species mixtures or that different switchgrass cultivars or cultivar mixtures responded differently (Fig. 3). This suggests that choosing a higher genotypic and/or species diversity will neither help or hinder production under moderate N fertilization, which is likely to be employed for perennial bioenergy crops given that producer profits are maximized at intermediate N inputs (Boyer *et al*., 2012). This finding is consistent with studies on other native perennial cropping systems in fertile soil (Jarchow & Liebman, 2013).

A meta‐analysis of switchgrass monocultures and switchgrass‐dominated mixtures found that mixtures of switchgrass and legumes generally performed well (Wang *et al*., 2010), presumably due to fertilization effects from N_2_ fixation. In our study, however, the treatment that included legumes (PR) had lower yields compared to switchgrass cultivar monocultures and lower diversity mixtures, whether unfertilized or fertilized, suggesting that there was no N fertilization effect due to the legumes (Fig. 3). This may have been due the low abundance of legumes in our mixture (7.5% of sown seed) and the poor establishment of these legumes (Fig. 2). Also, prairie mixture (PR) yields might have been lower because the broadleaf plants (e.g., the large composites) take up a large area when they are green but when they senesce (and are cut and baled), the leaves fall to the ground and are not harvested. Future studies on combined genotypic/species mixtures for biomass production might take advantage of a better understanding of optimal legume composition in biomass cropping systems that has emerged from recent work (Picasso *et al*., 2008; Johnson *et al*., 2010; Wang *et al*., 2010; Griffith *et al*., 2011).

Another potential impact of native biomass crops is gene flow from cultivars to native stands of the same species. The probability of such gene flow and its effects on native populations and associated communities are not yet understood (Kwit & Stewart, 2011). The inclusion of local ecopools in cultivar mixtures for biomass cropping systems could help conserve local genotypes. One aspect we considered here was whether local germplasm could be used effectively in biomass cropping systems. The results were mixed. The local switchgrass ecopool Southlow did not establish well, contributing little to the biomass in the early years of the experiment (Fig. 2) and yielding poorly in the early production years (Fig. 3). This is likely because Southlow, unlike Cave‐In‐Rock and Kanlow, was not intentionally selected for reduced dormancy and vigorous emergence. However, Southlow switchgrass yields continued to rise over the production years compared to Cave‐In‐Rock (which plateaued) while Kanlow decreased. This is consistent with the expectation that Southlow, from nearby southern Michigan, is well adapted to northeast Illinois (Casler *et al*., 2007; Grabowski *et al*., 2014). By the last production years (2013–2014), Southlow yields matched those of Cave‐In‐Rock and exceeded in those of Kanlow in the unfertilized treatment.

In many photoperiod sensitive species, including switchgrass, the use of low latitude‐adapted germplasm in high latitudes is known to dramatically increase biomass yields through delayed flowering (Casler *et al*., 2007; Rooney *et al*., 2007). Under favorable conditions, wet‐adapted germplasm also produce more biomass than dry‐adapted germplasm. Consistent with this expectation, we saw the lowest yields in the most northern and dry‐adapted cultivars, Dacotah switchgrass and Champ big bluestem (Fig. 4). The yields of Kanlow, the most southern and wet‐adapted switchgrass cultivar in our study, did achieve high yields in some years, but declined dramatically in 2013 and 2014. Cultivars from southern lowland gene pool would be poorly adapted to the northern Midwest climate (Casler *et al*., 2007; Grabowski *et al*., 2014). The unusually cold winter of 2013/2014 may have contributed to the decline of the Kanlow stands, as Kanlow stands have shown little survival just 175 km north of our field site (Casler, 2014). Overall, the balance between photoperiod effects and local adaptation effects seems to favor cultivars or mixtures from within the hardiness zone or one zone south of our site in the upper Midwest (e.g., Cave‐In‐Rock and Blackwell for switchgrass, and Rountree and Suther for big bluestem).

The composition of harvested biomass has important implications for crop management (e.g., balancing N‐P‐K removal and fertilization), biomass utilization (e.g., lignin/cellulose ratios or slagging and fouling) (Sims, 2003), and the environmental sustainability of the system (e.g., net energy inputs and GHG balance) (Downing *et al*., 2011; Singh *et al*., 2012; Bhandari *et al*., 2013). A previous study comparing four switchgrass and three big bluestem genotypes found the former having significantly higher lignin content, as determined by HPLC (Zhang *et al*., 2015). However, we saw no evidence of higher lignin in switchgrass overall in our comparison of seven switchgrass vs. seven big bluestem genotypes (Table 3). Given that we did observe among‐cultivar variation in lignin (Table 2 and 3), the lack of between‐species differences in lignin is not likely due to a lack of precision in the NIRS measurements, but the broader sampling of switchgrass and big bluestem germplasm in our study. Ash and N levels were similar for big bluestem and switchgrass, and big bluestem may have lower levels of minerals involved in corrosion/fouling (e.g., Cl, Ca, S; Table 2). Together with the evidence of high yield potential for big bluestem, these data suggest that big bluestem mixtures and monocultures warrant more consideration for biomass production systems.

**Table 3:** Biomass composition for switchgrass and bluestem cultivar monocultures (top) or different diversity levels (bottom). Values that are significantly different (*α *= 0.05) among cultivars or diversity levels are indicated by a different letter. For the cultivars, monocultures *n* = 4 and for the diversity levels *n* = 42 (1 cultivar) or *n* = 12 (2, 4, and 6 cultivars)

Species	Cultivar	Lignin	Cell	Hemi	Sugar	NFC	Fat	Ash	N	P	K	Mg	S	Ca	Na	Cl
Switchgrass	Blackwell	7.9ab	45.0ab	29.8ab	4.9bc	6.9b	1.4bc	5.3ab	0.54b	0.045b	0.15bc	0.17a	0.043b	0.29 cd	83.5a	0.095b
	CaveInRock	7.7ab	44.9ab	29.8ab	5.1b	7.0b	1.5bc	5.9ab	0.49b	0.060ab	0.15bc	0.16a	0.043b	0.31bcd	79.5a	0.065b
	Dacotah	7.5ab	39.7c	25.1c	6.5a	11.6a	2.3a	7.9a	1.03a	0.100a	0.30a	0.16a	0.075a	0.52a	123.0a	0.155a
	Forestburg	8.1a	44.5b	29.9ab	4.8bcd	5.9bc	1.4bc	6.4ab	0.57b	0.060ab	0.13c	0.16a	0.045b	0.40bc	92.5a	0.068b
	Kanlow	7.3ab	46.8ab	31.0a	4.9bc	4.5bc	1.5bc	6.5ab	0.55b	0.035b	0.14c	0.17a	0.043b	0.21d	92.2a	0.062b
	SouthlowSG	7.8ab	45.2ab	28.9ab	4.0 cde	6.3b	1.4bc	6.8ab	0.53b	0.053b	0.13c	0.14ab	0.045b	0.40bc	76.5a	0.092b
	Sunburst	8.1a	45.0ab	30.0ab	4.5bcd	5.9bc	1.3c	6.1ab	0.53b	0.055b	0.12c	0.17a	0.062ab	0.42ab	89.0a	0.070b
Big Bluestem	Bonanza	7.2ab	47.1ab	31.2a	3.3e	1.7c	1.6bc	7.3ab	0.58b	0.048b	0.17bc	0.13ab	0.040b	0.29d	107.2a	0.057b
	Champ	7.7ab	47.2ab	28.9ab	4.0cde	4.4bc	1.7b	6.5ab	0.56b	0.058b	0.19abc	0.12ab	0.043b	0.29d	83.0a	0.070b
	Epic	7.0b	46.1ab	29.8ab	4.0cde	4.9bc	1.7bc	7.0ab	0.53b	0.058b	0.28ab	0.10b	0.043b	0.26d	88.5a	0.083b
	Pawnee	7.9ab	45.3ab	30.2ab	4.5bcd	6.0bc	1.6bc	5.0b	0.64b	0.048b	0.18abc	0.14ab	0.043b	0.30cd	78.0a	0.087b
	Rountree	8.1a	48.0a	29.3ab	3.9de	3.4bc	1.5bc	5.8ab	0.58b	0.045b	0.21abc	0.10b	0.037b	0.24d	89.5a	0.068b
	SouthlowBB	7.8ab	48.2a	29.3ab	3.3e	2.5bc	1.4bc	7.1ab	0.54b	0.053b	0.16bc	0.11b	0.040b	0.27d	120.2a	0.058b
	Suther	8.0a	46.7ab	28.5b	5.3b	5.7bc	1.5bc	5.9ab	0.57b	0.045b	0.23abc	0.12ab	0.045b	0.25d	78.0a	0.068b

Species	No. of cultivars	Lignin	Cell	Hemi	Sugar	NFC	Fat	Ash	N	P	K	Mg	S	Ca	Na	Cl
Switchgrass	1	7.8ab	44.5d	29.2a	4.9a	7.0a	1.5a	6.4a	0.61a	0.058a	0.16b	0.16a	0.051a	0.37a	90.9a	0.087a
	2	8.0ab	45.2 cd	29.6a	4.8ab	5.7ab	1.4a	6.3a	0.56a	0.055ab	0.14b	0.16a	0.048ab	0.36ab	87.0a	0.083ab
	4	8.0ab	46.3abcd	29.6a	4.8ab	5.0ab	1.5a	5.8a	0.58a	0.048ab	0.14b	0.17a	0.045ab	0.32abc	119.3a	0.079ab
	6	7.9ab	45.8bcd	29.4a	4.8ab	5.7ab	1.4a	6.2a	0.56a	0.041b	0.14b	0.16a	0.043ab	0.34abc	88.8a	0.084ab
Big Bluestem	1	7.7b	47.0abc	29.6a	4.0 cd	4.1b	1.6a	6.4a	0.57a	0.050ab	0.20a	0.12c	0.041b	0.27c	92.1a	0.070ab
	2	8.2a	48.0a	28.9a	4.1bcd	3.6b	1.5a	6.0a	0.59a	0.055ab	0.19ab	0.12bc	0.040b	0.26c	91.4a	0.058b
	4	7.9ab	47.2abc	29.6a	4.1bcd	3.7b	1.6a	6.3a	0.56a	0.048ab	0.18ab	0.12c	0.040b	0.28bc	98.0a	0.065ab
	6	7.9ab	47.6ab	30.2a	3.8d	3.7b	1.5a	6.2a	0.57a	0.046ab	0.18ab	0.12bc	0.039b	0.27c	83.7a	0.057b
Mixture	2	7.8ab	47.0abc	29.6a	4.4abcd	4.3b	1.5a	7.0a	0.52a	0.041b	0.16b	0.14b	0.043ab	0.30bc	91.2a	0.075ab
	4	7.8ab	46.3abcd	30.3a	4.2bcd	4.8ab	1.4a	6.5a	0.58a	0.047ab	0.15b	0.14ab	0.043ab	0.32abc	85.8a	0.068ab
	6	8.0ab	46.4abc	29.4a	4.7abc	4.9ab	1.4a	6.0a	0.58a	0.043ab	0.14b	0.13bc	0.044ab	0.28bc	80.2a	0.069ab

Cell, cellulose; Hemi, hemicellulose; NFC, nonfiber carbohydrate.

The variation in feedstock composition introduced by higher diversity biomass cropping systems has been raised as a potential limitation of these systems (Adler *et al*., 2009). In our study, the prairie mixture (PR) did have significantly different composition for a number of variables, including notably higher lignin and ash than the Cave‐In‐Rock (CR) and Kanlow (KA) switchgrass monocultures (Table 2). In contrast, the switchgrass cultivar mixture (SG) and the big bluestem + switchgrass mixture (BB) had lignin levels similar to the Cave‐In‐Rock monoculture. Using genotypic mixtures of a single species, or simple mixtures of grasses, may be a way to exploit beneficial diversity effects while avoiding undesirable compositional effects of high species diversity. The low N composition of switchgrass had a role in its prioritization over leguminous crops for bioenergy research (Wright, 2007). Here, we observed no difference in biomass N among the plant treatments (Table 2), but this may be due to the small proportion of legumes in the harvested biomass and translocation of nutrients to belowground biomass prior to harvest. The nonlegume forbs, which did contribute substantially to harvested biomass, had no effect on N levels (Table 2). Biomass from N fertilized plots had significantly higher lignin and N than biomass from unfertilized plots (5% greater in both cases), indicating that the increase in yield upon N fertilization may go along with a reduction in feedstock quality.

Given the long breeding cycle for perennial outcrossing crops, the development of regionally optimized genotypes for perennial biomass production may take decades. Effectively implementing and interpreting multi‐environment and multiyear field trials in plant breeding programs remain a serious challenge even in major annual row crops (Cooper *et al*., 2014). Taken together, our findings on biomass yield and composition suggest that genotypic mixtures could be a useful strategy to increase and stabilize yields in biomass feedstock production systems, in parallel with crop improvement efforts. Further studies, especially in marginal production environments and on targeted genotypic mixtures, will help determine the potential value of genotypic mixtures to increase the sustainability and profitability of native perennial bioenergy cropping systems.
